# Production and partial characterization of extracellular amylase enzyme from *Bacillus amyloliquefaciens* P-001

**DOI:** 10.1186/2193-1801-2-154

**Published:** 2013-04-10

**Authors:** Promita Deb, Saimon Ahmad Talukdar, Kaniz Mohsina, Palash Kumar Sarker, SM Abu Sayem

**Affiliations:** 1 Department of Genetic Engineering and Biotechnology, Shahjalal University of Science and Technology, Sylhet, Bangladesh; 2 Microbial Biotechnology Division, National Institute of Biotechnology, Savar, Dhaka, Bangladesh

**Keywords:** *Bacillus amyloliquefaciens*, Extracellular amylase, Shake flask culture, Production optimization, Characterization

## Abstract

Amylases are one of the most important enzymes in present-day biotechnology. The present study was concerned with the production and partial characterization of extracellular amylase from *Bacillus amyloliquefaciens* P-001. The effect of various fermentation conditions on amylase production through shake-flask culture was investigated. Enzyme production was induced by a variety of starchy substrate but corn flour was found to be a suitable natural source for maximum production. Tryptone and ammonium nitrate (0.2%) as nitrogen sources gave higher yield compared to other nitrogen sources. Maximum enzyme production was obtained after 48 hrs of incubation in a fermentation medium with initial pH 9.0 at 42°C under continuous agitation at 150 rpm. The size of inoculum was also optimized which was found to be 1% (v/v). Enzyme production was 2.43 times higher after optimizing the production conditions as compared to the basal media. Studies on crude amylase revealed that optimum pH, temperature and reaction time of enzyme activity was 6.5, 60°C and 40 minutes respectively. About 73% of the activity retained after heating the crude enzyme solution at 50°C for 30 min. The enzyme was activated by Ca^2+^ (relative activity 146.25%). It was strongly inhibited by Mn^2+^, Zn^2+^ and Cu^2+^, but less affected by Mg^2+^ and Fe^2+^.

## Background

Microbial enzymes are widely used in industrial processes due to their low cost, large productivity, chemical stability, environmental protection, plasticity and vast availability (Burhan et al. 2003 ; Mishra & Behera 2008 ). *Bacillus* species such as *Bacillus subtilis*, *Bacillus amyloliquefaciens* and *Bacillus licheniformis* are used as bacterial workhorses in industrial microbial cultivations for the production of a variety of enzymes as well as fine biochemicals for decades. A large quantity (20-25g/l) of extracellular enzymes has been produced and secreted by the various *Bacillus* strains which have placed them among the most significant industrial enzyme producers. The estimated value of world market is presently about US$ 2.7 billion and is estimated to increase by 4% annually through 2012. Detergents (37%), textiles (12%), starch (11%), baking (8%) and animal feed (6%) are the main industries, which use about 75% of industrially produced enzymes ( Harwood 1992 ; Ferrari et al. 1993 ; Schallmey et al. 2004 ; Das et al. 2011 ). Amylases are among the most important enzymes and are of great significance for biotechnology, constituting a class of industrial enzymes having approximately 25-30% of the world enzyme market (Azad et al. 2009 ; Rajagopalan & Krishnan 2008 ). Initially the term amylase was used originally to designate an extracellular enzymes capable of hydrolyzing α-1,4-glucosidic linkages in polysaccharides containing three or more 1,4-α-linked glucose units. The enzyme acts on starches, glycogen and oligosaccharides in a random manner, liberating reducing groups. These enzymes are found in prokaryote as well as in eukaryotic organisms. They are widely distributed in microbial, plant and animal kingdoms. In the present day scenario, amylases have a great commercial value in biotechnological applications ranging from food, fermentation, textile to paper industries. These uses have placed greater stress on increasing amylase production and search for more efficient processes (Aehle & Misset 1999 ; Lin & Hsu 1997 ; Wolfgang 2007 ). For the maximum enzyme production, medium optimization is a prime step for its commercial usage.

The present work describes the effects of culture conditions on amylase production in batch experiments in shake flasks and under controlled conditions in a laboratory incubator. In this study, we show that enzyme synthesis is affected by carbon and nitrogen sources and maximal activity is attained with inorganic than organic nitrogen sources. The optimum enzyme production by the bacterial isolate was found at 42°C, whereas maximum enzyme activity was observed at 60°C. The enzyme was activated by Ca^2+^ (relative activity 146.25%). It was strongly inhibited by Mn^2+^, Zn^2+^ and Cu^2+^, but less affected by Mg^2+^ and Fe^2+^.

## Results and Discussion

**Figure 1:**
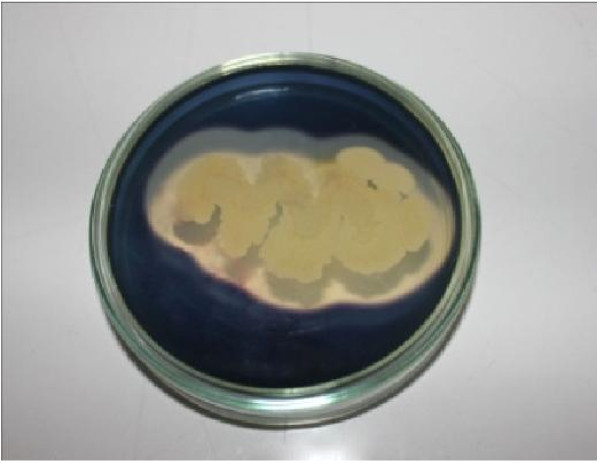
**Zone of clearance due to the hydrolysis of starch.** Screening of bacterial isolate for capability of amylase production was done by starch hydrolysis plate assay method. Bacterial isolate was streaked as a line on the starch agar plate and plates were incubated at 37°C for 24 h & 48 h.

The genus Bacillus produces a large variety of extracellular enzymes, of which amylases are of particularly considerable industrial importance (Swain et al. 2006 ). Present study deals with the production condition optimization and partial characterization of crude extracellular amylase produced by *Bacillus amyloliquefaciens* P-001. *Bacillus amyloliquefaciens* P-001 were able to hydrolyze starch showing zone of hydrolysis around the colonies on agar medium supplemented with soluble starch (Figure 1).

Therefore, further studies on enzyme production in shake-flask cultures were carried out using *Bacillus amyloliquefaciens* P-001. The organism was used for extracellular amylase production in shake-flask culture using basal medium (0.1% KH_2_PO_4,_ 0.25% Na_2_ HPO_4,_ 0.1% NaCl, 0.2% (NH_4_)_2_SO_4_, 0.005% MgSO_4_ .7H_2_O, 0.005% CaCl_2_, 0.2% tryptone and 1% soluble starch, pH 6.5) (Bose & Das 1996 ) for 48 hrs hours of incubation at 37°C and enzyme activity was obtained 35.0 U/ml. To enhance the production of enzyme various parameters associated with the production of amylase were studied in the medium used for the enzyme production. Optimization of culture conditions is very important for maximum microbial growth and enzyme production by microorganisms (Kathiresan & Manivannan 2006 ). Among the physical and chemical parameters, optimum temperature, pH range, carbon and nitrogen sources are the most important for enzyme production by microbes (Bose & Das 1996 ; Gupta et al. 2003 ).

Among physical parameters, pH of the growth medium plays an important role in enzyme secretion. The pH range observed during the growth of microbes also affects product stability in the medium (Banargee & Bhattacharya 1992 ). Most of the earlier studies revealed an optimum pH range between 6.0 and 7.0 for the growth of bacterial strains and enzyme production (Gupta et al. 2003 ; Kundu et al. 1973 ; Castro et al. 1992 ). Previous studies have revealed that fungi required slightly acidic pH and bacteria required neutral pH for optimum growth (Gangadharan et al. 2006 ). So, the effect of initial pH on the production of amylase by *Bacillus amyloliquefaciens* P-001 was investigated at different pH (6.0-9.5). The activity of the enzyme was obtained at slightly alkaline pH 9.0. But, the final pH of the medium (initial pH 9.0) of shake flask fermentation was 7.5. At neutral pH, the results were moderate and at acidic pH the enzyme activity was extremely low (Table 1). It might be due to the fact that the enzyme was inactive in the acidic medium (Castro et al. 1993 ). (Nusrat & Rahman 2007 ) reported that, α-amylase production at pH 7.0 by the *Bacillus amyloliquefaciens* was maximum. Another study conducted by O. El- Tayeb (2007 ) showed that alpha amylase production by *Bacillus amyloliquefaciens* (strain 267CH) in fermentor was highest at pH 6.0. (El-Tayeb et al. 2007 ).

**Table 1:** **Effect of culture conditions for extracellular amylase production from*Bacillus amyloliquefaciens*P**-**001 in shake**-**flask cultivations**

Culture condition	Amylase activity (U/ml) (Mean ± SE)	Relative activity (%)	Total soluble protein (mg/ml) (Mean ± SE)
Initial pH			
6.0	17.25±0.53	48.13	0.87±0.01
6.5	22.65±0.58	63.20	1.45±0.03
7.0	27.71±0.45	77.32	1.03±0.04
7.5	28.09±0.93	78.38	2.76±0.03
8.0	28.92±0.10	80.69	3.01±0.04
8.5	31.01±0.94	86.52	2.69±0.02
9.0	35.84±0.17	100.00	2.84±0.03
9.5	25.39±0.31	70.84	2.73±0.02
Incubation temperature (°C)			
32	25.75±0.53	46.88	3.34±0.05
35	37.14±0.89	67.61	2.35±0.06
37	39.20±0.19	71.36	2.09±0.05
40	53.85±0.40	98.03	1.79±0.02
42	54.93±0.18	100.00	1.18±0.03
45	30.33±0.59	55.22	2.40±0.04
Incubation period (hr)			
24	10.49±0.19	38.24	4.15±0.04
48	27.43±0.08	100.00	3.97±0.07
72	26.28±0.21	95.81	3.84±0.05
96	24.04±0.07	87.64	3.23±0.06
Inoculums volume (%)			
1.0	39.25±0.20	100.00	-
1.5	35.92±0.27	91.52	-
2.0	34.46±0.17	87.80	-
2.5	31.18±0.27	79.44	-
3.0	30.97±0.18	78.90	-
3.5	28.38±0.18	72.31	-

Data represent as mean ± standard error (SE) for three replicates.

Temperature is a vital environmental factor which controls the growth and production of metabolites by microorganisms and this is usually varied from one organism to another (Banargee & Bhattacharya 1992 ; Kumar & Takagi 1999 ). Bacterial amylases are produced at a much wider range of temperature. *Bacillus amyloliquefaciens*, *B*.*subtilis*, *B*. *licheniformis* and *B*. *stearothermophilus* are among the most commonly used *Bacillus* sp. reported to produce α-amylase at temperatures 37–60°C (Mendu et al. 2005 ; Mielenz 1983 ; Syu & Chen 1997 ; Mishra et al. 2005 ). A wide range of temperature (35-80°C) has been reported for optimum growth and α-amylase production in bacteria (Burhan et al. 2003 ; Castro et al. 1992 ; Prakash et al. 2009 ; Lin et al. 1998 ). In present study, for the determination of optimum temperature for enzyme production, the fermentation was carried out at different temperatures (32 to 45°C). Enzyme production was gradually increased with increasing temperature and maximum enzyme production was observed at 42°C (Table 1). The optimum range for enzyme production was 40-42°C. Nusrat & Rahman (2007 ) reported that, α-amylase production was maximum at temperature 37°C by the *Bacillus amyloliquefaciens*. Haq et al. ( 2010 ) reported that, the better activity of α-amylase in stirred fermentor with working volume of 4.5 L was at 37°C in 48 h by using randomly induced mutant strain of *Bacillus amyloliquefaciens EMS6*.

From the time course study in shake culture, it was found that the rate of enzyme production was increased with the increase in the fermentation period and reached its maximum activity after 48 hour incubation (Table 1). The total protein content obtained was 3.97 mg/ml at that time. A prolonged incubation time beyond 48 hour did not increase the enzyme production. These findings are similar to the result reported by Haq et al. ( 2010 ). A similar result was also found by Asgher et al. ( 2007 ) studied on *Bacillus subtilis*, Kaur & Vyas (2012 ) in case of *Bacillus* sp. DLB 9 and Riaz (et al. 2003 ) in case of *Bacillus subtilis*. It might be due to the accumulation of other by products in the medium Riaz et al. ( 2003 ). Efficient induction might not occur until the stationary phase has been reached and the available carbon source was reduced (Huang et al. 2003 ; Wanderley et al. 2004 ). But, Abate et al. ( 1999 ) reported that the production of α-amylase by *Bacillus amyloliquefaciens* starts at the beginning of the exponential growth phase reaching the maximum level after 24 hour and after that, α-amylase level decreased drastically probably due to the accumulation of high level of protease activities concomitant with the sporulation process at the end of the exponential growth phase. Similar findings have been reported on *Bacillus amyloliquefaciens* Hillier et al. ( 1997 ) and *Bacillus sp*. ANT-6 Burhan et al. ( 2003 ).

The volume of inoculum plays an important role in the fermentation of enzymes Lin et al. ( 1998 ). In our study, 1% inoculum induced the maximum amylase production (Table 1). As the inoculum level was further increased, the production of enzyme was gradually decreased. It may be due to the fact that at high concentration of inoculum level, the bacteria grow rapidly and the nutrients present in the media were insufficient to overcome the growth of bacteria. Thus, the production of amylase was affected at higher concentration of inoculum. Our findings are in a good agreement with Riaz et al. ( 2003 ).

**Figure 2:**
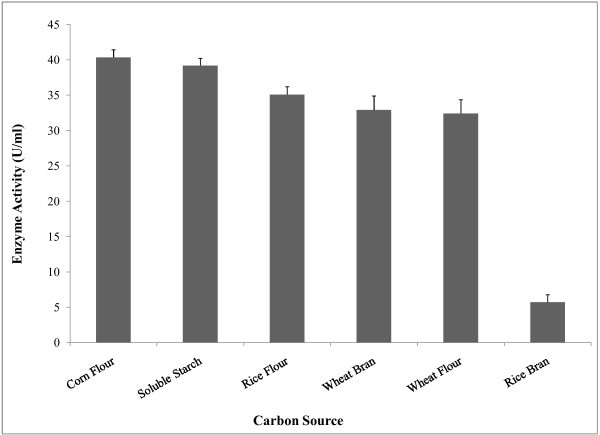
**Effect of carbon sources on amylase production by*Bacillus amyloliquefaciens*P**-**001.** The effect of different carbon sources on enzyme production was investigated by using 1% inoculums (w/v) in 100 ml basal medium. The fermentation was carried out at 37°C at 150 rpm for 48 hrs. Absorbance was measured at 540 nm with spectrophotometer and Enzyme activity was presented on the y axis and carbon sources was on x axis. Bars represent means ± standard deviations for three replicates.

Natural sources could serve as economical and readily available raw material for the production of valuable enzymes. Agricultural wastes are being used for liquid fermentation to reduce the cost of fermentation media. These wastes consist of different carbon sources are necessary for the growth of microorganisms (Haq et al. 2005 ; Swamy & Seenayya 1996 ; Djekrif-Dakhmouche et al. 2005 ). The nature and amount of carbon sources in culture media are important factor for the production of extracellular amylase Akcan (2011 ). It was found that α-amylase production was maximum when starch was used as the carbon source (Gupta et al. 2003 ; Sumrin et al. 2011 ; Bandyopadhyay et al. 1993 ; Lin et al. 1994 ; Narang & Satyanarayana 2001 ; Bozic et al. 2011 ; Sexana et al. 2007 ). Biosynthesis of the enzyme took place not only in the presence of starch but also with other carbon sources. Our study showed that the production of enzyme was highest when corn flour was used as carbon source in the basal media (Figure 2). Besides, rice flour and wheat bran showed moderate effects on enzyme synthesis. Earlier studies reported that, complex substrates induce higher amylase production Sexana et al. ( 2007 ).

**Figure 3:**
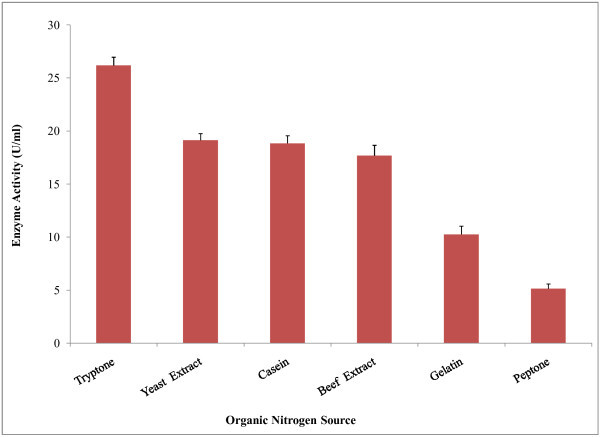
**Effect of organic nitrogen source on amylase production by*Bacillus amyloliquefaciens*P**-**001.** Different organic nitrogen sources (0.2% w/v) in 100 ml of basal medium were used for the experiment and the medium was incubated at 37°C at 150 rpm for 48 h in a rotary shaking incubator. Enzyme activity was presented on the y axis and organic nitrogen sources was on x axis. Bars represent means ± standard deviations for three replicates.

**Figure 4:**
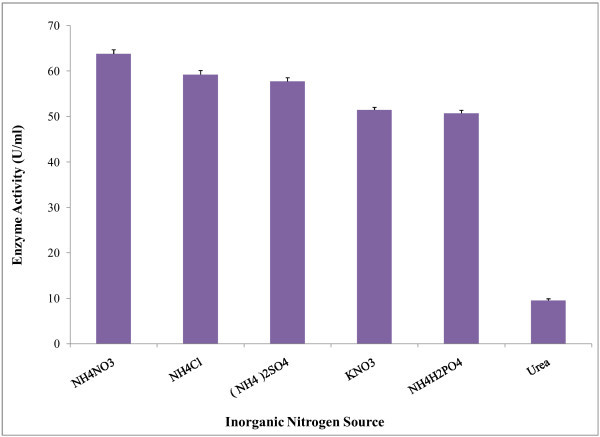
**Effect of inorganic nitrogen source on amylase production by*Bacillus amyloliquefaciens*P**-**001.** To determine the effect of inorganic nitrogen sources on enzyme production different inorganic nitrogen sources were used (0.2% w/v) in 100 ml of basal medium. The fermentation was carried out at 37°C at 150 rpm for 48 h. Bars represent means ± standard deviations for three replicates.

The nature and relative concentration of different complex nitrogenous sources in the growth medium are both important in the synthesis of amylase. Lower levels of nitrogen and also excess nitrogen are equally detrimental causing enzyme inhibition Sharma et al. ( 2012 ). The influence of organic nitrogen sources on amylase production was determined. Among the different organic nitrogen sources tested, tryptone (0.2%) was found to be a good nitrogen source for amylase production from *Bacillus amyloliquefaciens* P-001 (Figure 3). In fact, tryptone has been reported to be the best nitrogen source for amylase production Okalo et al. ( 1996 ). In present study, yeast extract, casein and beef extract also showed stimulating effects on amylase synthesis. It has been reported that yeast extract also served as good organic nitrogen source for α-amylase synthesis from *Bacillus amyloliquefaciens* (Sharma et al. 2012 ; Magee & Kosaric 1987 ). Similarly, casein was reported to be a good nitrogen source for α-amylase production from *B*. *subtilis* IP 5832 Bozic et al. ( 2011 ). It was observed that inorganic nitrogen sources gave comparatively higher yields than organic nitrogen sources. In present study, the enzyme production was increased when ammonium nitrate used as inorganic nitrogen source in the culture media. According to Coleman & Elliott (1962 ) ammonium salts were stimulators of B. *subtilis* amylase synthesis. Our findings are in a good agreement with the findings of these studies (Figure 4). It has also been reported that, ammonium nitrate and sodium nitrate were the best nitrogen sources for maximum amylase production (Kundu et al. 1973 ; Mahmood & Rahman 2008 ). Ammonium chloride, ammonium sulphate showed stimulating effects on amylase production. It has been found that α-amylase production by *B*. *subtilis* DM-03 was maximum when ammonium chloride as the nitrogen source Das et al. ( 2004 ). Swain et al. ( 2006 ) studied on *Bacillus subtilis* reporting that, urea inhibited α-amylase activity which is similar to our findings.

**Figure 5:**
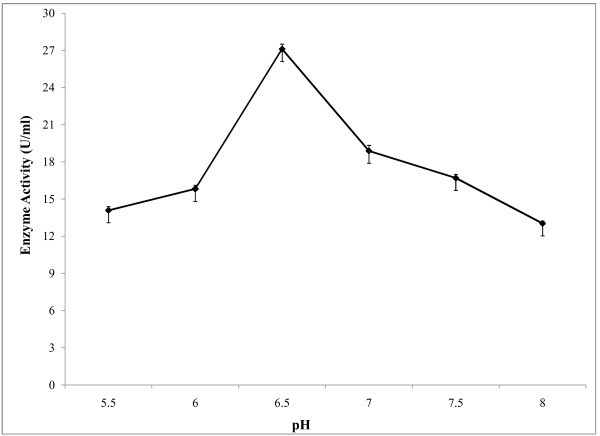
**Effect of pH on enzyme activity.** For determination of optimum assay pH of the enzyme reaction, 0.05 M Na_2_HPO_4_-NaH_2_PO_4_ buffer was used. The reaction was carried out for 10 min at 50°C in a shaking water. The enzyme activity was measured and the results are presented on graph. Bars represent means ± standard deviations for three replicates.

**Figure 6:**
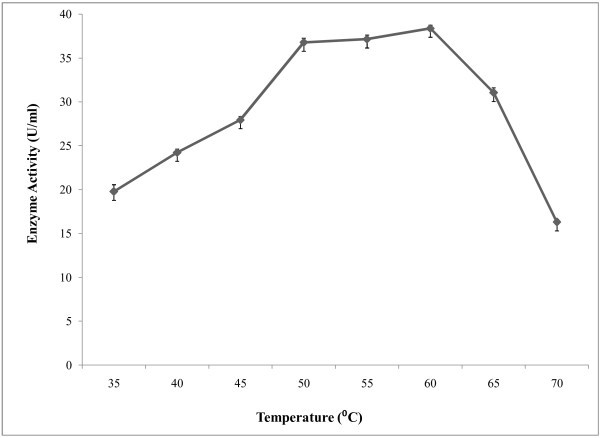
**Effect of temperature on enzyme activity.** To study the effect of temperature on enzyme reaction activity, the enzyme reaction was carried out at different temperatures for 10 min in a shaking water bath and results are presented on graph. Bars represent means ± standard deviations for three replicates.

**Figure 7:**
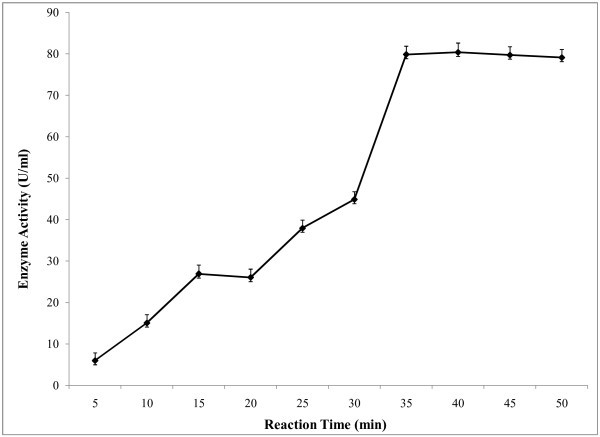
**Effect of reaction time on enzyme activity.** To investigate the optimum reaction period of the enzyme solution, reaction was carried out using 0.05 M sodium phosphate buffer (pH 6.5) at 50°C in a water bath at different time intervals and the enzyme activity was then measured. The results are presented on graph. Bars represent means ± standard deviations for three replicates.

**Figure 8:**
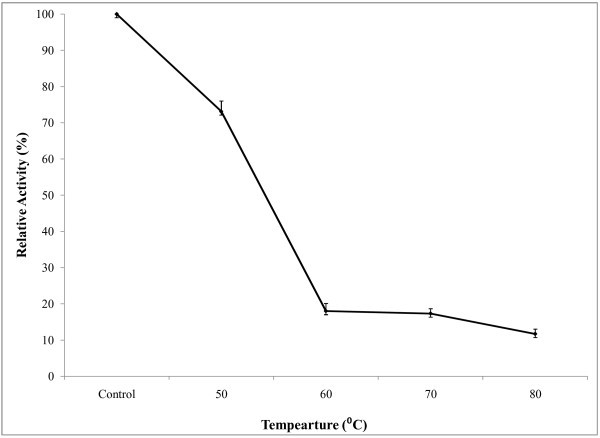
**Thermal stability of enzyme.** For the determination of thermostability of amylase, 1 ml of sodium phosphate buffer (pH 6.5) and 1 ml of enzyme solution containing test tubes were heated at different temperatures for 30 minutes in a shaking water bath. Then enzyme activity of the heat treated enzymes was then measured and the results are presented on graph. Bars represent means ± standard deviations for three replicates.

**Figure 9:**
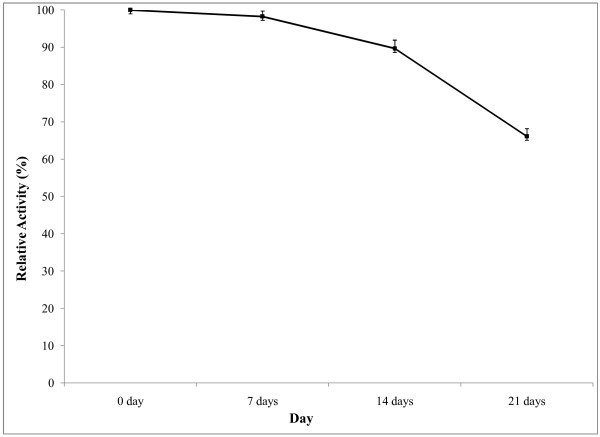
**Storage stability of enzyme.** To determine the storage stability of amylase enzyme, crude enzyme solution was stored at room temperature and the activity was measured at 7 days interval over a month by standard assay method described previously. The results are presented on graph. Bars represent means ± standard deviations for three replicates.

A pH range from 5.5-8.0 was used to study the effect of pH on amylase activity (Figure 5) and optimum pH was found at 6.5. In the alkaline pH range, the activity was lower. The effect of temperature on enzyme activity was assayed at different temperatures ranging from 35-70°C at optimum pH. The results showed that enzyme activity was increased with temperature and it showed highest activity at temperature 60°C (Figure 6). Above 60°C temperature activity was also decreased. For determination of optimum reaction time, enzyme assay was carried out at different reaction time ranging from 5–50 minutes at constant temperature and pH. It was observed (Figure 7) that, the enzyme exhibited its maximum activity at 40 min of reaction time. Aygan et al. ( 2008 ) in 2008 reported that enzyme obtained from *Bacillus* sp. AB68 was active in a broad temperature range between 20 and 90°C, with an optimum of 50°C. Stability of the enzyme is of great importance for the economy of their industrial application. In case of thermostabilty, the enzyme was pre incubated at different temperatures for 30 min and then enzyme was assayed. The results (Figure 8) showed that the enzyme activity was retained 73% after heating at 50°C for 30 min. After this time the activity was decreased drastically and enzyme was completely inactivated when heated at 80°C. Thus, the results concluded that the crude enzyme is moderately temperature stable. It is therefore worthwhile to consider means to stabilize the enzyme under storage conditions. Temperature is an important limiting factor for storage of enzymes. In our study, enzyme was stored at room temperature for 21 days. But, the room temperature was moderately suitable for the storage of this enzyme in considering commercialization and industrial application, thus causing the rapid reduction of enzyme activity. Only 66% of the activity retained at room temperature after 21 days (Figure 9).

**Figure 10:**
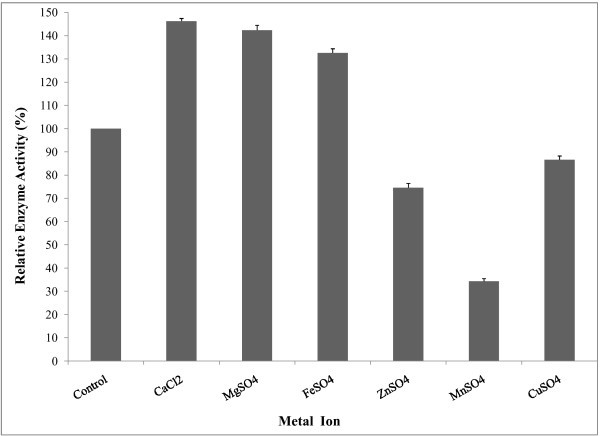
**Effect of metal ions on enzyme activity.** For determining the effect of metal ions on amylase activity, enzyme assay was performed after pre-incubation of the enzyme with various metal ions each at a concentration of 2 mM at 50°C for 30 min. The results are presented on graph. Bars represent means ± standard deviations for three replicates.

Most of amylases are known to be metal ion-dependent enzymes, namely divalent ions like Ca^2+^, Mg^2+^, Mn^2+^, Zn^2+^, Fe^2+^ etc. Pandey et al. ( 2000 ). The effect of metal ions on amylase activity was measured in the presence of various metal ions at a concentration of 2 mM. Activities of enzyme were stimulated in the presence of Ca^2+^, Mg^2+^ and Fe^2+^ ions (Figure 10). On the other hand, a strong inhibitory effect was observed in the presence of Cu^2+^, Zn^2+^ and Mn^2+^. Results suggest that amylase did not require any metal ions for catalytic activity except Ca^2+^ and was activated (relative activity 146%) by calcium. Tonkova (1991 ) and Chung et al. ( 1995 ) stated that, addition of Ca^2+^ ion is often significant for production and stability of amylase of many *Bacillus* spp. According to Gupta et al. ( 2003 ), α-amylase contain at least one Ca^2+^ ion and affinity of Ca is much stronger than that of other metal ions.

## Conclusions

The cultural conditions and composition of media for optimal production of amylase by *B*. *amyloliquefaciens* P-001 has been developed in this study. Enzyme synthesis was affected by carbon and nitrogen sources and maximal activity was attained with inorganic than organic nitrogen sources. The optimum enzyme production by the bacterial isolate was found at 42°C, whereas maximum enzyme activity was observed at 60°C which could make the enzyme from *B*. *amyloliquefaciens* P-001 more suitable for future use in various industries. It can be concluded that, *B*. *amyloliquefaciens* P-001 can be a potential producer of extracellular amylase which could find applications in industry. Due to the importance of these findings, further studies need be carried on in order to commercialize the production process.

## Methods

### Microorganism

The bacterial culture *Bacillus amyloliquefacience* P-001 was obtained from the Microbial Biotechnology Division, National Institute of Biotechnology, Ganakbari, Savar, Dhaka. It was maintained on nutrient agar medium. The organism was maintained at 4°C in refrigerator for routine laboratory use. For the long term preservation, the log phage growth bacteria were maintained in 15% glycerol broth at -20°C.

### Plate assay method

The *Bacillus* isolates were tested for amylase activity by employing zone clearing technique Atlas et al. ( 1995 ) using starch agar medium. The inoculated plates were incubated at 37°C for two days. After incubation, the zone of hydrolysis of starch was detected by flooding the plates with iodine solution. The development of blue colour indicated the presence of starch, while the areas around the hydrolytic bacteria appeared clear.

### Preparation of seed culture

Vegetative inoculums were used in the present studies. Fifty millilitre of inoculums medium containing nutrient broth 13 g/l, pH 7.4 ± 0.2 was transferred to a 100 ml conical flask and cotton plugged. It was sterilized in an autoclave at 15 lbs/inch^2^ pressure at 121°C for 20 min. After cooling to room temperature, a loopful of freshly grown culture was aseptically transferred to it. The flask was incubated overnight at 37°C and 150 rpm in a rotary shaking incubator.

### Enzyme production in shake flask cultures

The enzyme production was carried out in the basal Asgher et al. ( 2007 ) medium containing 0.1% KH_2_PO_4,_ 0.25% Na_2_ HPO_4,_ 0.1% NaCl, 0.2% (NH_4_)_2_SO_4_,0.005% MgSO_4_ .7H_2_O, 0.005% CaCl_2_, 0.2% tryptone and 1% soluble starch. 1 ml of 24 hours grown inoculums were cultivated in 250-ml Erlenmeyer flasks containing 100 ml of medium with an initial pH 7.0. The cultures were shaken at 150 rpm in a orbital shaker incubator at 37°C for at least 48 h unless otherwise stated. After the incubation, the fermented broth was centrifuged in a refrigerated centrifuge machine at 8000 rpm for 15 minutes at 4°C.

### Enzyme assay

Amylase was determined by using soluble starch, 1% (w/v), as substrate in 0.05 M Sodium phosphate buffer (pH 6.5) essentially according to Gomes et al. ( 2001 ). The reaction mixture containing 1.8 ml substrate solution and 0.2 ml suitably diluted enzyme solution was incubated at 50°C for 10 min. The reaction was stopped by adding 3 ml dinitrosalicylic acid (DNS). The reducing sugar released was determined by the method of Miller (1959 ). The absorbance was measured at 540 nm with spectrophotometer (Jenway 6305, USA). One unit (U) of enzyme activity is defined in all cases as the amount of enzyme releasing 1 μg of reducing sugar as maltose per minute, under assay conditions.

### Soluble protein estimation

Extracellular soluble protein in culture filtrate was estimated by the Lowry’s method using bovine serum albumin (BSA) used as Standard Lowry et al. ( 1951 ). 2 ml of analytical reagent was added to 0.2 ml suitably diluted test samples (enzyme solution). The mixture was mixed well and allowed to stand for 10 min at 50°C. Then 0.2 ml of the folin-ciocalteau reagent was added and shaken to mix well and incubated at room temperature for about 30 min. Optical density of the reaction mixture was measured at 600 nm, against a blank prepared with 0.2 ml buffer. A standard curve was constructed with each experiment using bovine serum albumin as a known protein. The amount of the soluble protein was calculated from the standard curve of as mg protein per ml of test samples.
